# First isolation and identification of *Cystobasidium calyptogenae* from the oral samples of an elderly patient presenting with angular cheilitis

**DOI:** 10.1186/s40001-022-00671-6

**Published:** 2022-03-27

**Authors:** Alexandria Sonia Karajacob, Joanne Pei En Goh, Thomas George Kallarakkal, Sun Tee Tay

**Affiliations:** 1 Department of Medical Microbiology, Faculty of Medicine, Universiti Malaya, Kuala Lumpur, Malaysia; 2 Department of Oral and Maxillofacial Clinical Sciences, Faculty of Dentistry, Universiti Malaya, Kuala Lumpur, Malaysia; 3 Oral Cancer Research and Coordinating Centre (OCRCC), Faculty of Dentistry, Universiti Malaya, Kuala Lumpur, Malaysia

**Keywords:** Angular cheilitis, *Candida* yeasts, *Cystobasidium calyptogenae*, Oral samples

## Abstract

**Background:**

Angular cheilitis, an infection mainly caused by *Candida* yeasts, is featured by the appearance of inflammatory lesions at the bilateral corners of the mouth, particularly in patients with poor oral hygiene, ill-fitting dentures and old age. The first isolation of an atypical yeast, *Cystobasidium calyptogenae,* from oral samples of a patient presenting with angular cheilitis is discussed in this study.

**Case presentation:**

Angular cheilitis was diagnosed in a 60-year-old denture-wearing woman who presented with an irritation fibroma on her right lower buccal sulcus over the premolar region. Primary cultures of her oral swab and oral rinse samples grew a pure culture of an uncommon yeast strain resembling *Rhodotorula* sp. Sequence analysis of the yeast internal transcribed spacer (ITS) gene region and D1D2 domain showed highest similarity (99.6% and 100%, respectively) to *C. calyptogenae* CBS 9125 type strain. Following 2 weeks of treatment with miconazole/fusidic acid and mouthwash, the oral lesion showed improvement with less erythema. *C. calyptogenae* was not isolated from the patient’s oral samples upon repeat sampling.

**Conclusion:**

This is the first report on the isolation of *C. calyptogenae* from human oral samples. The ability of *C. calyptogenae* to grow at 37 °C and the fact that it was the only yeast species isolated from the patient’s oral samples suggests its pathogenic potential and possible involvement in angular cheilitis. The ubiquitous nature of the *Cystobasidium* yeast is believed to increase the likelihood of opportunistic infections among immunocompromised individuals. As *Cystobasidium* is phenotypically indistinguishable from *Rhodotorula,* an emerging opportunistic pathogen, surveillance using molecular identification in clinical settings is essential in providing accurate diagnosis and treatment of uncommon yeast infections.

## Background

Angular cheilitis (angular stomatitis; cheilosis; perleche) is characterized by burning sensations, soreness, redness, bleeding and fissures at the bilateral corners of the oral cavity [1]. The disease has been linked to various underlying risk factors including poor oral hygiene, long-term and ill-fitting dentures, inadequate vitamin B consumption, protein and trace minerals (zinc or iron) deficiency, immunocompromised states (acquired immunodeficiency syndrome, diabetes, chemotherapy), prolonged antibiotic use and old age [1, 2]. The inflammatory erythematous lesions of angular cheilitis are commonly associated with the *Candida* yeasts and occasionally, *Staphylococcus aureus* and β-hemolytic streptococci [3, 4]*. Candida albicans* and *S. aureus* are acknowledged as co-infectors in 60—75% cases of angular cheilitis [1].

*Cystobasidium* (Langerheim) Neuhoff (1924) is a mycoparasitic yeast associated with coprophilous ascomycetes. The yeast forms soft, smooth and light orange to pinkish colonies upon culturing on mycological agars [5–12]. To date, 21 *Cystobasidium* species have been described, i.e., C*. fimetarium, C. minutum, C. slooffiae, C. calyptogenae, C. pinicola, C. laryngis, C. benthicum, C. pallidum, C. lysinophilum, C. portillonensis, C. oligophagum, C. alpinum, C. psychroaquaticum, C. rietchieii, C. tubakii, C. ongulense, C. keelungensis* [6–10], *C. halotolerans* [11], *C. iriomotense C. raffinophilum* and *C. terricola* [13]. Various habitats and diverse ecologies, i.e., supraglacial sediments [9], aquatic environments [10, 14–18], soil [8, 19–21] and phylloplanes [7, 22–26] have been associated with *Cystobasidium* yeasts. Previously classified in the *Rhodotorula minuta* clade [7], members of this genus have not been described as commensal organisms or human pathogens.

*C. calyptogenae* is ovoidal to elongate with polar budding appearance [7, 9]. It was first isolated from *Calyptogena* sp., a species of giant white clam, at a depth of 1156 m from Sagami Bay, Japan, and was initially named *Rhodotorula calyptogenae* [5]. The detection of *C. calyptogenae* from food sources [27], human household items [28], pets [29], soil, plant materials [30–32] and marine ecosystems [5, 33–35] suggests its high adaptability to various environments.

The emerging agents of opportunistic fungal infections may impact the treatment and management of oral diseases in elderly and immunocompromised individuals. This study reports the isolation and characterization of an uncommon yeast species, *C. calyptogenae,* from the oral swab and rinse samples of an elderly patient presenting with angular cheilitis.

## Case presentation

A 60-year-old denture-wearing Malaysian woman was referred to the Oral Medicine Clinic, Universiti Malaya, in February 2020, with the complaint of a painless swelling on her lower right cheek for a month. She also had moderate xerostomia, with a Clinical Oral Dryness Score of five [36, 37]. Upon examination, the patient was partially edentulous with no obvious facial swelling or palpable lymph nodes. Slight erythema was noted at the bilateral angle of her mouth, suggestive of angular cheilitis. A non-tender, non-indurated lump measuring 0.5 × 0.5 cm resembling an irritation fibroma, most likely caused by an ill-fitting denture, was observed on her lower right buccal sulcus over the premolar region.

An oral swab was collected from the bilateral angle of her mouth for Gram staining and cultured for *Candida* yeasts on Brilliance Candida agar™ (BCA) (Oxoid, UK). An expectorated oral rinse sample collected from gargling 20 ml of saline was also obtained and centrifuged at 9600 revolutions per minute at 4 ºC for 10 min. The pellet (100 μl) was cultured for isolation of *Candida* yeasts. Gram-stained smear of the oral swab showed presence of scanty Gram-positive and Gram-negative bacteria and epithelial cells. Blastoconidia or hyphal filaments were not observed. Primary cultures from the oral swab and oral rinse samples grew dark blue colonies along the initial streak lines on BCA upon incubation at 37 °C after 48 h of incubation. The colonies were subcultured onto fresh BCA and Sabouraud’s dextrose agar (SDA) plates for incubation at room temperature and 37 °C. Smooth, mucoidal, and light orange or beige colonies were observed on SDA at both temperatures. Similar colonies were observed on BCA at room temperature; however, dark blue colonies were observed on BCA at 37 °C. The yeast growth was generally faster at room temperature compared to 37 ºC on both agar media. Gram stain examination of a pure culture showed round-to-oval yeast morphology. The yeast isolated was assigned as *C. calyptogenae* strain D1.

The yeast was identified to the species level through polymerase chain reaction (PCR) amplification and sequence determination of the internal transcribed spacer (ITS) gene and D1/D2 domain of the large subunit (LSU) ribosomal DNA (rDNA) of the yeast. The freeze–thaw method as described by Silva et al. [38] was used to extract yeast DNA. Two sets of primers, ITS1 (5ʹ-TCC GTA GGT GAA CCT GCG G-3ʹ) and ITS4 (5ʹ-TCC TCC GCT TAT TGA TAT GC-3ʹ) [39], and primer pair NL1 (5ʹ- GCA TAT CAA TAA GCG GAG GAA AAG-3ʹ) and NL4 (5ʹ-GGT CCG TGT TTC AAG ACG G-3ʹ) were used for species identification. The PCR reaction mixture (25 μl) contained 2 μl (16 ng) of DNA extract, 1 μM of each primer, 12.5 μl of exTen 2 × PCR Master Mix (1st Base, Singapore). The PCR amplification conditions included 5 min denaturation at 95 ºC for 1 cycle, followed by 35 cycles of 95 ºC for 1 min, 52 ºC for 1 min, 72 ºC for 1 min and one final extension step of 72 ºC for 10 min. The nucleotide sequences of the amplified products were determined by a commercial sequencing provider (Apical Scientific, Malaysia), using forward and reverse PCR primers. The sequences were assembled on the Biological Sequence Alignment Editing (Bioedit) software (RRID: SCR_007361) and searched for the highest sequence similarity using the GenBank Basic Local Alignment Search Tool (BLASTn) (RRID: SCR_004870) against the National Centre for Biotechnology Information (NCBI) nucleotide database (RRID: SCR_004860). A phylogenetic tree was constructed on the Molecular Evolutionary Genetics Analysis (MEGA) software (RRID: SCR_000667) using ITS gene sequences of *C. calyptogenae* strains retrieved from the GenBank database. *Erythrobasidium hasegawianum* strain CBS 8253 (AF444522) was used as an outgroup.

**Fig. 1:**
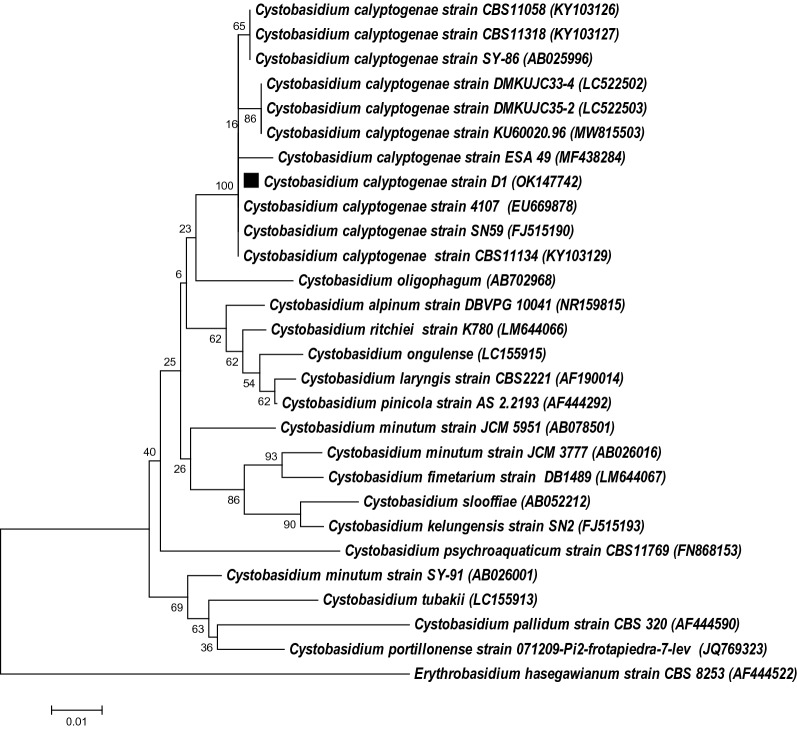
Neighbour-joining phylogenetic tree (Jukes–Cantor model) constructed based on ITS gene sequences of *Cystobasidium* species (refer to Table 1 for source and details of *C. calyptogenae* strains; black square = phylogenetic position of *C. calyptogenae* strain D1). Bootstrap values generated from 1000 replications are represented on the node of every branch

The yeast D1/D2 domain sequence (GenBank accession no. OK147747) was 100% (558/558 nucleotides) similar to the *C. calyptogenae* CBS 9125 type strain, which was first isolated from a giant white clam (AB025996) [5]. Other strains demonstrating 100% sequence similarity include *C. calyptogenae* strain 4107 (EU669877) [33], which was isolated from seawater in Taiwan, and strain CBS 11058 from a culture collection [40]. Meanwhile, the yeast ITS sequence (GenBank accession no. OK147742) exhibited 100% (463/463 nucleotides) similarity to *C. calyptogenae* strain 4107 (EU669877) [33], and CBS 11134 (KY103129) [40] but 99.5% (2 nucleotide difference) to *C. calyptogenae* CBS 9125 type strain. Figure 1 shows the phylogenetic tree constructed based on ITS sequences of various *Cystobasidium* reference strains. Strain D1 was clustered with *C. calyptogenae* CBS 9125 type strain, and other known *C. calyptogenae* strains in the same branch with high bootstrap value (100%). Based on phylogenetic analysis, the identity of strain D1 was thus confirmed as *C. calyptogenae.*

The patient was given topical treatment with 2% miconazole and fusidic acid, once daily, for the management of angular cheilitis. Mouthwash (Oral-7) was provided to improve oral dryness. The patient was also scheduled for the construction of a new lower denture to increase the vertical dimension of the mouth to prevent recurrence of angular cheilitis. The clinical condition of the patient improved during a follow-up visit 2 weeks later, with mild erythema observed on the affected area. Repeat oral swab and oral rinse cultures were negative for *C. calyptogenae*. However, a mixed growth of yeasts (*Trichosporon asahii, Candida dubliniensis,* and *Candida parapsilosis*) was noted in the oral rinse sample. Similar treatment was thus continued for another 2 weeks and the patient was placed on an open appointment that required her to come to the clinic only if the symptoms persisted.

## Discussion and conclusions

*C. calyptogenae* was the only yeast isolated from both primary cultures of oral swab and rinse samples of the patient investigated in this study. The ability of *C. calyptogenae* to multiply at 37 °C suggests its virulence potential to infect human hosts [41], and further strengthens our speculation on its pathogenic potential. Currently, there are 12 published studies describing the isolation of *C. calyptogenae* from a variety of environmental sources [5, 27–35, 42, 43] (Table 1). The presence of *C. calyptogenae* in homemade fermented rice water [27], dishwashers [28], plant and soil samples [30–32], solidified radioactive waste disposal sites [43], marine ecosystems (including sea surface microlayer, underlying seawater, corals, clams and crabs) [29, 34, 42], as well as the external ear canal of a cat [35], may indicate possible human exposure through various points of environmental sources [44]. Given the ubiquitous nature of this *Cystobasidium* yeast, the likelihood of opportunistic infections may increase among immunocompromised individuals.

**Table 1:** Source and details of *C. calyptogenae* strains reported in the literature

Strain (GenBank accession no.)	Host/sample	Geographical region	References
Type strain JCM 10899/SY-86 (AB025996)	*Calyptogena* sp. (giant clam)	Sagami bay, Japan	Nagahama et al*.(*2003) [5]
Strain 4107 (EU669878)	Seawater	Tainan, Taiwan	Tien et al*.* (2008) [33]
Strain EXF-6094 (JF766634)^†^	Dishwasher	Martinique, France	Zalar et al*.* (2011) [28]
Strain IPM32-15 (AB726383)^†^	Soil and plant samples	Japan	Takashima et al*.* (2012) [32]
Strain RT1^†^	Solidified radioactive waste	Lanyu, Taiwan	Li et al*.* (2015) [43]
Strains CBS 11058 (KY103126), Strain CBS 11318 (KY103127), Strain CBS 11134 (KY103129)	Centraalbureau voor Schimmelcultures database	–	Vu et al*.* (2016) [40]
Strain ESA 49 (MF438284)	Umbu fruit	Petrolina, Brazil	Gava et al*.* (2018) [30]
Strain DMKU-CP557 (LC430175)^†^	Corn (*Zea mays* Linn.) leaves	Suphan Buri, Thailand	Into et al*.* (2020) [31]
Strain DMKU JC9-1 (LC431010)^†^, Strain DMKUJC33-4 (LC522502), Strain DMKUJC35-2 (LC522503)	Corals (*Klyxum* sp., *Cladiella* sp.)	Chonburi, Thailand	Kaewkrajay et al*.* (2020) [34]
Strain R68^†^	Homemade fermented rice water	Phitsanulok, Thailand	Wongwigkarn et al*.* (2020) [27]
Strain *C. calyptogenae* (MW815503)	Cat/external ear canal	Bangkok, Thailand	Niae et al*.* (2021) [29]
Strain 5890^†^	Crab (*Xenograpus testudinatus*)	Kueishan Island, Taiwan	Shaumi et al*.* (2021) [35]
Strain D1 (OK147742)	Human/oral swab and rinse	Malaysia	This study

^†^ITS gene was not available for comparison

The observation of dark blue colonies of *C. calyptogenae* strain D1 on BCA at 37 ºC has not been described before in literature. *Rhodotorula mucilaginosa,* a close relative of *Cystobasidium* yeast, has been reported to grow salmon-pink colonies on BCA at 37 ºC [45]. Generally, pink to orange colonies of *Cystobasidium* spp. on SDA at 20–22 ºC [7, 8, 10–12] are due to the production of carotenoid pigment, torularhodin [46, 47]. Higher temperatures have been reported to reduce carotenoid production [12]. Hence, the development of dark blue yeast colonies on BCA at 37 ºC could be due to accumulated effects of reduced carotenoid production and enzymatic reactions with chromogens in BCA.

Among members of the genus *Cystobasidium*, *C. fimetarium* has been reported to be mycoparasitic towards ascomycetes such as *Lasiobolus equinus*, *Saccobolus violaceus* and *Thelebolus crustaceus* [48]. Mycoparasitism was similarly reported through the biocontrol ability of *C. calyptogenae* (MF438284) from a native umbú (*Spondias tuberosa)* fruit [30]. In vitro and in vivo antagonistic effects of the yeast were demonstrated against multiple fungal pathogens (i.e., *Lasiodiplodia theobromae*, *Fusicoccum aesculli*, *Neofusicoccum parvum* and *Colletotrichum dianesei*) affecting postharvest of mangoes. Biotechnological potentials have also been recognized in closely related species such as *C. psychroaquaticum,* for its extracellular enzyme and carotenoid production [12]. Therefore, further exploration of *C. calyptogenae* strain D1 as a mycoparasitic biocontrol agent may help provide an alternative approach for the treatment of oral fungal diseases.

An increase in the incidence of oral candidiasis has been recently reported in elderly patients [49, 50]. This study documents the first isolation and identification of *C. calyptogenae*, an environmental yeast, from the oral samples of an elderly patient presenting with angular cheilitis. In recent years, several clinically relevant red-color pigmented *Rhodotorula* species have been reported to cause opportunistic infections such as meningitis, endocarditis, fungemia, central venous catheter infections, keratitis [51], as well as non-healing oral ulcers [52]. As *Cystobasidium* and *Rhodotorula* yeasts are difficult to differentiate phenotypically [53], molecular screening is necessary in providing accurate identification for the surveillance of these opportunistic fungal infections. Further research is essential to unravel the mechanisms of infection and mycoparasitism of *C. calyptogenae* in the human oral cavity, in addition to various underlying factors that may increase the risk of angular cheilitis [1].

## Data Availability

The sequences generated and analyzed in this study were deposited into GenBank database, under the accession numbers OK147742 and OK147747.

## Abbreviations

Bioedit: Biological Sequence Alignment Editing
BCA: Brilliance Candida agar™
*C. calyptogenae*: *Cystobasidium calyptogenae*
*C. albicans*: *Candida albicans*
BLASTn: GenBank Basic Local Alignment Search Tool
ITS: Internal transcribed spacer
LSU: Large subunit
Min: Minute
MEGA: Molecular Evolutionary Genetics Analysis
PCR: Polymerase chain reaction
rDNA: Ribosomal DNA
SDA: Sabouraud’s dextrose agar
*S. aureus*: *Staphylococcus aureus*
