# Correction: First isolation and identification of *Cystobasidium calyptogenae* from the oral samples of an elderly patient presenting with angular cheilitis

**DOI:** 10.1186/s40001-022-00884-9

**Published:** 2022-12-06

**Authors:** Alexandria Sonia Karajacob, Joanne Pei En Goh, Thomas George Kallarakkal, Sun Tee Tay

**Affiliations:** 1 Department of Medical Microbiology, Faculty of Medicine, Universiti Malaya, Kuala Lumpur, Malaysia; 2 Department of Oral and Maxillofacial Clinical Sciences, Faculty of Dentistry, Universiti Malaya, Kuala Lumpur, Malaysia; 3 Oral Cancer Research and Coordinating Centre (OCRCC), Faculty of Dentistry, Universiti Malaya, Kuala Lumpur, Malaysia

**Correction: European Journal of Medical Research (2022) 27:48** https://doi.org/10.1186/s40001-022-00671-6

In the original version of the article [1], the statement in the Funding information section was incorrectly published. The corrected funding information is provided below. The original article has been corrected.

**Funding**

The work is supported financially by the Ministry of Higher Education Malaysia via Fundamental Research Grant Scheme (FRGS/1/2019/SKK11/UM/01/1).
